# High frequency of clonal IG and T-cell receptor gene rearrangements in histiocytic and dendritic cell neoplasms

**DOI:** 10.18632/oncotarget.13058

**Published:** 2016-11-03

**Authors:** Wenting Huang, Tian Qiu, Linshu Zeng, Bo Zheng, Jianming Ying, Xiaoli Feng

**Affiliations:** ^1^ Department of Pathology, National Cancer Center, Cancer Hospital, Chinese Academy of Medical Sciences and Peking Union Medical College, Beijing, China

**Keywords:** histiocytic sarcoma, Langerhans cell histiocytosis, Langerhans cell sarcoma, interdigitating dendritic cell sarcoma, follicular dendritic cell sarcoma, Pathology Section

## Abstract

The 2008 World Health Organization (WHO) diagnostic criteria of histiocytic and dendritic cell neoplasms from hematopoietic and lymphoid tissues no longer required the absence of clonal B-cell/T-cell receptor gene rearrangements. It is true that the clonal B-cell/T-cell receptor gene rearrangements have been identified in rare cases of histiocytic and dendritic cell neoplasms, such as those with or following lymphoma/leukemia or in some sporadic histiocytic/dendritic cell sarcomas, but the clonal features of such group of tumor are still not clear. Here we investigated the clonal status of 33 samples including Langerhans cell histiocytosis (LCH), Langerhans cell sarcoma (LCS), follicular dendritic cell sarcoma (FDCS), interdigitating dendritic cell sarcoma (IDCS) and histiocytic sarcoma (HS). Among them, twenty-eight cases were sporadic without current or past lymphoma/leukemia. Three cases were found with a past history of T-cell lymphoma, one case was followed by extraosseous plasmacytoma, and one case was found with diffuse large B-cell lymphoma (DLBCL). Our results showed that there was a high frequency of clonal IG and T-cell receptor gene rearrangements in these cases. Notably, 4 cases of LCH and 2 cases of FDCS showed both B and T cell receptor gene rearrangements concurrently. One case of FDCS synchronous with DLBCL showed identical clonal IGH in both tumor populations and clonal TCRβ in FDCS alone. No matter if the presence of clonal receptor gene rearrangements was associated with the tumor origin or tumorigenesis, it might serve as a novel tumor marker for developing target therapy.

## INTRODUCTION

Histiocytic and dendritic cell neoplasms are rare among the tumors of hemapoietic and lymphoid tissues, and only account for less than 1% of hematolymphoid tumors presenting in lymph nodes [1]. They can also occur in extranodal sites, such as skin, liver, bone and soft tissue [2–6]. Histiocytic and dendritic cell neoplasms are consisted of a group of tumors including histiocytic sarcoma (HS), Langerhans cell histiocytosis/Langerhans cell sarcoma (LCH/LCS), interdigitating dendritic cell sarcoma (IDCS) and follicular dendritic cell sarcoma (FDCS). The histogenetic origins of these tumors are from myeloid-derived macrophages, myeloid-derived dendritic cells, and stromal-derived dendritic cells, which separately are the precursors of histiocytes, Langerhans cells and a part of the interdigitating cells, and follicular dendritic cells.

The recognition of tumors has changed in the last two decades. In 2001 the World Health Organization (WHO) classification of tumors of the hematopoietic and lymphoid tissues defined histiocytic and dendritic cell neoplasms as a tumor without clonal B or T cell receptor gene rearrangements [7]. But in 2008 [1], the WHO classification described rare cases of HS, IDCS, and FDCS that have been reported to have antigen receptor gene rearrangements. The reason for this modification was that clonal B or T cell receptor gene rearrangements had been identified in a few cases of HS and LCH, which was reported, previously, simultaneously or subsequently to a non-Hodgkin's lymphoma, such as precursor B or T-lymphoblastic leukemia/lymphoma [8–11], follicular lymphoma [12] and myeloid leukemia/sarcoma [13, 14]. In addition, in the investigation by Chen [15] et al, 39% cases of sporadic histiocytic/dendritic cell sarcomas without either a past history or a concurrent diagnosis of any type of lymphoma showed clonal IGH (±IGK) gene rearrangements. This provided evidence that there was a high frequency of clonal immunoglobulin receptor gene rearrangements in sporadic histiocytic/dendritic cell sarcoma.

In order to get further information about the molecular characteristics of histiocytic and dendritic cell neoplasms and to learn more about the nature of the correlation between such group of tumors and lymphoma/leukemia, we investigated the clonal status of 33 samples that included LCH, LCS, FDCS, IDCS, and HS. Twenty eight of these cases were sporadic without a history or concurrent with lymphoma/leukemia, while three cases were with a past history of T-cell lymphoma, one case was followed extraosseous plasmacytoma, and one case was concomitant with diffuse large B-cell lymphoma (DLBCL).

## RESULTS

### Clinical features

All patients enrolled in this study ranged in age from 8 to 74 years (median, 42 years). Among 33 patients, 20 were male and 13 were female. The demographic and clinical characteristics of LCH, LCS, FDCS, IDCS and HS were listed in Table 1, respectively. The most common primary site of LCH was bone. And in our study all 5 cases of HS occurred in lymph node. As shown in the Table 1, tonsil was the most common extranodal site of FDCS.

**Table 1:** Clinical Characteristics of all samples

Characteristics		LCH	LCS	FDCS	IDCS	HS
Total		13	2	11	2	5
Age (range, years)		8-74	30-43	28-66	26-47	17-47
	mean age	33	37	49	37	36
Gender						
	Male	8	2	6	0	4
	Female	5	0	5	2	1
Primary Site						
	Lymph node	3	1	3		5
	Extranodal site					
	Bone	7				
	Tosil			5		
	Larnyx			1		
	Skin		1			
	Lung	2				
	Adrenal gland	1			1	
	Liver			1		
	Stomach			1	1	

### Morphological features

**Figure 1:**
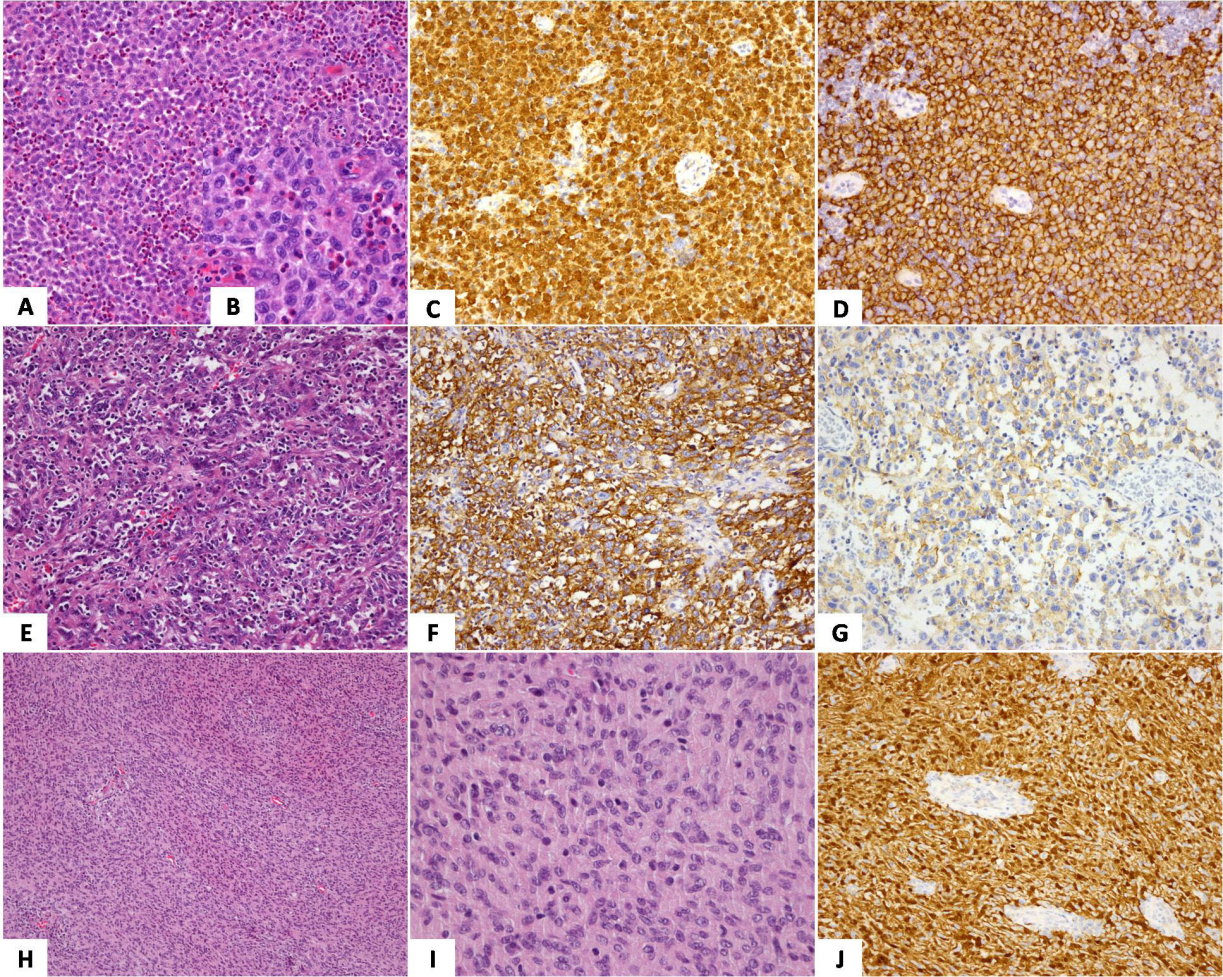
LCH (A-C) **A.** A variable number of eosinophils were admixed with tumor cells (H&E×100); **B.** Tumor cells had grooved, folded nuclei with inconspicuous nucleoli (H&E×400); **C.** Staining was both nuclear and cytoplasmic with S100 (×200); **D.** Tumor cells were diffuse strong positive for CD1α(×200). FDCS (E-G): **E.** Tumor cells had indistinct cytoplasmic outlines, and cytologic atypia was present (H&E×200); **F.** Tumor cells were positive for CD21 (×200); **G.** Staining for CD35 were also positive (×200). IDCS (H-J): **H.** Sheets of spindled cells formed a whorled pattern (H&E×40); **I.** The cytoplasm of tumor cells were abundant and had an indistinct border (H&E×400); **J.** Staining for S100 was strongly positive in the tumor cells (×200).

Thirteen cases of LCH contained a large number of cells with the characteristics of grooved, folded or lobulated nuclei. A variable number of eosinophils, neutrophils, histiocytes and small lymphocytes were scattered among tumor cells. Compared to LCH, 2 cases of LCS showed considerable pleomorphism, nuclear atypia, and high mitotic activity. Furthermore, eosinophils were decreased so that in some area they were difficult to be found. In LCH and LCS, tumor cells were typically stained positive for CD1α and S-100 (Figure 1A-1D).

FDCS were predominantly comprised of spindle to ovoid cells arraying storiform, whorls or sheet patterns with oval or elongated nuclei. Tumor cells had indistinct cell borders and a moderate amount of eosinophilic cytoplasm. Intranuclear inclusions were occasionally seen. And variable number of small lymphocytes were scattered among tumor cells. Tumor cells were stained positive for CD21, CD23 and CD35 (Figure 1E-1G).

The tumor cells of the two cases of IDCS were mainly polygonal, ovoid shaped. And they arranged in a diffuse pattern with numerous intermingled lymphocytes. The cytoplasm was abundant and the cyto-membrane was unclear. The nuclei were vesicular and the nucleoli were conspicuous and varied in size from small to large. Immunohistochemically, tumor cells showed S-100 positive, but CD1α negative (Figure 1H-1J).

### Clonal rearrangement analysis

Table 2 summarizes our major findings. In 28 sporadic cases, 6 of 11 (54.5%) cases of LCH, 7 of 9 (77.8%) cases of FDCS and 3 of 5 (60.0%) cases of HS were identified with clonal rearrangements, of which 4 of 11 (36.4%) cases of LCH and 2 of 9 (22.2%) cases of FDCS showed both B and T cell receptor gene rearrangements concurrently. One of 11 cases (9.1%) of LCH, 3 of 9 (33.3%) cases of FDCS, 1 of 5 (20.0%) case of HS showed only B cell receptor gene rearrangements. One of 11 cases (9.1%) of LCH, 2 of 9 (22.2%) cases of FDCS and 2 of 5 (40.0%) cases of HS showed only T cell receptor gene rearrangements as well. No clonal rearrangements were detected in any of LCS and IDCS samples.

**Table 2:** The Clonal Rearrangement Results of Positive Cases

Case No.	Clonal Rearrangement			
	IgH	IgK	TCRβ	TCRγ
**LCH**				
8	N	N	N	N
9*	+	−	−	−
11*	−	−	+	−
16	−	−	+	+
19,21,26	−	+	+	−
20	−	+	−	−
23	+	+	+	+
**FDCS**				
1,10,25*	−	+	−	−
13,15	−	−	+	+
22	−	+	+	−
24	+	+	−	−
29	+	+	−	+
33	+	−	+	−
**HS**				
2	−	+	−	−
6,18	−	−	+	−

* 9: with a history of cutaneous T-cell lymphoma; 11: with a history of anaplastic large cell lymphoma; 25: with a history of mycosis fungoides.

**Figure 2:**
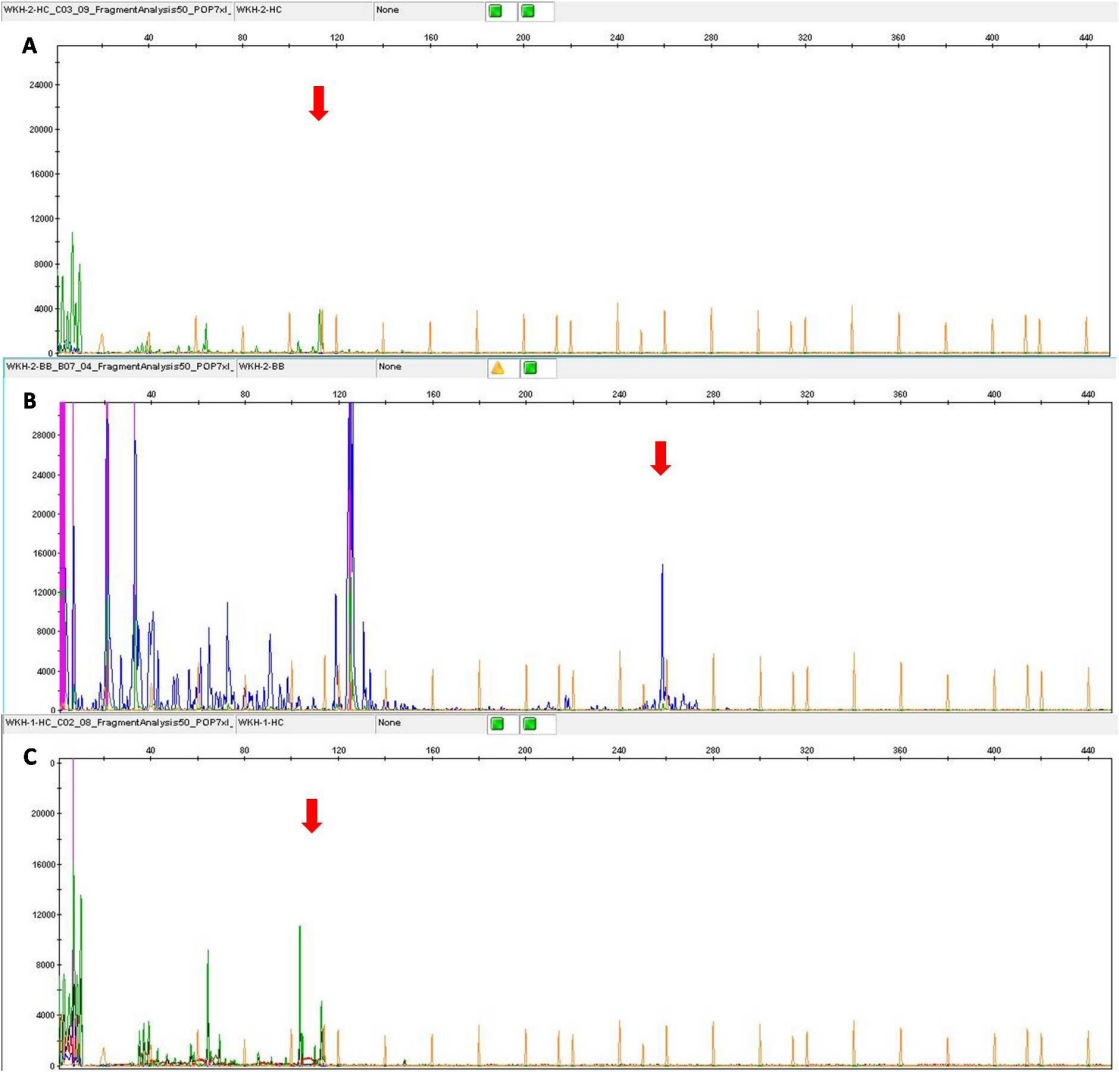
The case of FDCS concurrent with DLBCL FDCS showed clonal IGH **A.** and TCRβ gene rearrangement **B.**, and DLBCL showed clonal clonal IGH gene rearrangement **C.**

One case of LCH following cutaneous T-cell lymphoma was identified with IGH gene rearrangement. A second case of LCH with a past history of anaplastic large cell lymphoma showed TCRβ gene rearrangement. One case of FDCS following mycosis fungoides showed IGK gene rearrangement. A second case of FDCS concurrent with DLBCL showed clonal IGH in both populations and clonal TCRβ in FDCS alone (Figure 2). While one case of HS followed by extraosseous plasmacytoma showed neither T-cell nor B-cell receptor rearrangement.

The detailed results are shown in Table 2.

## DISCUSSION

Histiocytic and dendritic cell neoplasms are a group of rare tumors originating from common precursors in the hematopoietic and lymphoid system. Due to their extremely low incidence, the recognition of such group of neoplasms is limited, especially in their genetic characteristics. In the past twenty years, some cases of LCH or HS or FDCS reported in patients who also had T or B-cell lymphoma/leukemia [8, 9, 12] or myeloid sarcoma/leukemia [14]. And these cases showed that the two different hematopoietic neoplasms in the same patient had identical genetic abnormalities, indicating a clonal relationship between the two different types of neoplasms. Among these rare reported cases, most of them were LCH/LCS or HS developing in patients with synchronous or metachronous lymphoblastic leukemia/lymphoma of T- or B-cell lineage [8, 10, 16, 17]. So it was speculated that tumor cells in lymphoblastic neoplasms are precursor cells, which might have more lineage plasticity than other mature lymphoid tumor cells [18]. Moreover, although histiocytic and dendritic cell neoplasms occurring in patients with mature B-cell lymphoma were extremely rare, a few cases have successively been reported. The related mature B-cell lymphoma included follicular lymphoma (FL), extranodal marginal zone B-cell lymphoma of mucosa-associated lymphoid tissue (MALT lymphoma) and chronic lymphocytic leukemia/small lymphocytic lymphoma (CLL/SLL) [12, 18–20]. Therefore, based on these results of reported cases, the description of genetics of HS, IDCS and FDCS was modified in 2008 WHO classification, in which it was written that rare cases were reported to show antigen receptor gene rearrangements, suggesting a process of transdifferentiation [1]. After that, several cases of histiocytic and dendritic cell neoplasms were also reported to have the same B or T cell receptor gene rearrangements and chromosomal aberrations as the associated lymphoid neoplasms [21–29]. However, a high frequency of clonal immunoglobulin receptor gene rearrangements was also detected in sporadic histiocytic/dendritic cell sarcomas without either a past history or a concurrence of any type of lymphoma. It was thought that a large subset of histiocytic/dendritic cell sarcomas might have inherited B-cell genotypes and originally be derived from committed B-cell progenitors [15]. Hence, there were two main speculations about the clonal receptor gene rearrangements in histiocytic and dendritic cell neoplasms. One was the tumor lineage switching: it was said that one tumor cell might transdifferentiate into another [8, 27, 30]. And the other was the two tumor lineage, which arises by malignant transformation of a common stem cell.

In order to further understand the genetic characteristics of such group of tumors, we detected not only clonal IGH/IGK but also TCRβ/TCRγ gene rearrangements in 33 cases of histiocytic or dendritic cell neoplasms. These 33 cases were divided into two groups. One group was the sporadic cases having no history of any type of lymphoma/leukemia. And another group was associated with lymphoma. In the sporadic cases, our results showed clonal IGH/IGK or TCRβ/TCRγ gene rearrangements in more than half of LCH, FDCS and HS, which was similar to the previous findings [15]. But compared with that, we also detected TCRβ/TCRγ gene rearrangements, and found there was a high frequency of T-cell receptor gene rearrangements in histiocytic and dendritic cell neoplasms as well. Thus, our findings demonstrated that clonal receptor gene rearrangements were a common phenomenon in this series of hemopoietic tumors. In addition, 4 of 11 (36.4%) LCH cases and 2 of 9 (22.2%) FDCS cases in our study showed both B and T cell receptor gene rearrangements concurrently in all 28 sporadic cases. More interestingly, one case of FDCS concurrent with DLBCL showed the same clonal IGH gene rearrangement in both the two distinct tumor populations and clonal TCRβ in only FDCS tumor cells. All these findings seemed to provide an evidence to support the view that histiocytic and dendritic cell neoplasms were developed from lymphoid -committed progenitors, and two different types of neoplasms might be derived from a common precursor and differentiate towards two different directions under pathological conditions. Meanwhile, experimental research based on mouse model also showed that epidermal Langerhans cells were generated from mouse lymphoid-committed CD4^low^ precursors with the capacity to differentiate into T cells, B cells, CD8^+^ lymphoid dendritic cells, and natural killer cells. So the author hypothesized that Langerhans cells belong to the lymphoid lineage [31].

But it was worthy to note that immunoglobulins had been detected in a variety of cancer cells [32], such as breast, colon, lung, liver, cervical and oral cancers, and also in acute myeloid leukemia [33]. Besides, it was revealed that these immunoglobulins may involve in cell survival and promote tumor growth and migration [34, 35]. Therefore, we speculated that clonal receptor gene rearrangements detected in histiocytic and dendritic cell neoplasms might not necessarily mean that these neoplasms were derived from a lymphoid precursor. Instead, they might play an important role in tumorigenesis of histiocytic and dendritic cell neoplasms.

In short, for unknown reason, there was a high frequency of clonal IG and T-cell receptor gene rearrangements in histiocytic and dendritic cell neoplasms. It may serve as a novel tumor marker for developing target therapy.

## MATERIALS AND METHODS

### Case selection

Thirty three surgical resected or excisional biopsy samples, including 13 cases of LCH, 2 cases of LCS, 11 cases of FDCS, 2 cases of IDS and 5 cases of HS were collected at National Cancer Center/Cancer Hospital, Chinese Academy of Medical Sciences (CICAMS) in Beijing, between Jul 2004 and Jan 2016. The clinical parameters of these patients were recorded, including age at the diagnosis, gender, primary site and follow-up data, especially to make sure whether the patient developed a lymphoma/leukemia. Of all the 33 cases, one case of LCH was diagnosed following a 14-year history of cutaneous T-cell lymphoma, but the detailed subtype was unclear. Another case of LCH was diagnosed over one year after anaplastic large cell lymphoma. One case of FDCS was arisen following a 13-year history of mycosis fungoides. And one case of HS was followed by extraosseous plasmacytoma one year later. And one case of FDCS was concurrent with DLBCL in the same lymph node. The remaining 28 cases including 11 cases of LCH, 2 cases of LCS, 9 cases of FDCS, 2 cases of IDCS and 4 case of HS were all sporadic, not previously, simultaneously or subsequently to a lymphoma/leukemia.

All samples were formalin-fixed paraffin embedded (FFPE) and reviewed to confirm for the diagnosis based on hematoxylin and eosin (H&E)-stained sections and immunohistochemical staining according to the 4^th^ edition of the WHO Classification of Tumors of Haematopoietic and Lymphoid Tissues by two experienced hematopathologists (H.W. and F.X.).

### B-cell and T-cell clonality analysis

The case of FDCS concurrent with DLBCL and other cases involving lymph node were first microdissected on the paraffin sections to separate the tumor tissue and population avoiding contamination. Genomic DNA was extracted from paraffin-embedded tumor tissues using QIAamp^®^ DNA Mini Kit (Qiagen, Germany), according to the manufacturer's instructions. Quality and concentration of the DNA samples were examined by NanoDrop (Thermo). BIOMED-2 polymerase chain reaction (PCR) was performed to analyze the clonal expansion of T and B cells using IdentiClone™ T and B-Cell Clonality Assays (Invivoscribe, America). T-cell clonal expansion was detected by analysis of TCRβ and TCRγ gene rearrangement and B-cell clonal expansion by IGH and IGK gene rearrangement. The PCR products were analyzed using capillary electrophoresis on an ABI 3500XL genetic analyzer (Applied Biosystems). Appropriate positive and negative controls were included in all experiments.

## References

[1] Grogan TM, Pileri SA, Chan JKC, Swerdlow SH, Campo E, Harris NL (2008). Histiocytic and dendritic cell neoplasms. WHO Classification of Tumours of Haematopoietic and Lymphoid Tissues.

[2] Pileri SA, Grogan TM, Harris NL, Banks P, Campo E, Chan JK, Favera RD, Delsol G, De Wolf-Peeters C, Falini B, Gascoyne RD, Gaulard P, Gatter KC (2002). Tumours of histiocytes and accessory dendritic cells: an immunohistochemical approach to classification from the International Lymphoma Study Group based on 61 cases. Histopathology.

[3] Alayed K, Medeiros LJ, Patel KP, Zuo Z, Li S, Verma S, Galbincea J, Cason RC, Luthra R, Yin CC (2016). BRAF and MAP2K1 mutations in Langerhans cell histiocytosis: a study of 50 cases. Hum Pathol.

[4] Morren MA, Vanden BK, Vangeebergen L, Sillevis-Smitt JH, Van Den Berghe P, Hauben E, Jacobs S, Van Gool SW (2016). Diverse Cutaneous Presentations of Langerhans Cell Histiocytosis in Children: A Retrospective Cohort Study. Pediatr Blood Cancer.

[5] Ansari J, Naqash AR, Munker R, El-Osta H, Master S, Cotelingam JD, Griffiths E, Greer AH, Yin H, Peddi P, Shackelford RE (2016). Histiocytic sarcoma as a secondary malignancy: pathobiology, diagnosis, and treatment. Eur J Haematol.

[6] Pang J, Mydlarz WK, Gooi Z, Waters KM, Bishop J, Sciubba JJ, Kim YJ, Fakhry C (2016). Follicular dendritic cell sarcoma of the head and neck: Case report, literature review, and pooled analysis of 97 cases. Head Neck.

[7] Weiss LM, Grogan TM, Muller-Hermelink HK, Jaffe ES, Harris NL, Stein H (2001). Histiocytic and dendritic cell neoplasms. Pathology and Genetics of Tumours of Haematopoietic and Lymphoid Tissues.

[8] Feldman AL, Berthold F, Arceci RJ, Abramowsky C, Shehata BM, Mann KP, Lauer SJ, Pritchard J, Raffeld M, Jaffe ES (2005). Clonal relationship between precursor T-lymphoblastic leukaemia/lymphoma and Langerhans-cell histiocytosis. Lancet Oncol.

[9] Brown AF, Fan H, Floyd JR, Henry JM, Higgins RA (2015). Primary Central Nervous System Histiocytic Sarcoma Arising After Precursor B-Cell Acute Lymphoblastic Leukemia. J Neuropathol Exp Neurol.

[10] Feldman AL, Minniti C, Santi M, Downing JR, Raffeld M, Jaffe ES (2004). Histiocytic sarcoma after acute lymphoblastic leukaemia: a common clonal origin. Lancet Oncol.

[11] Pastor-Jané L, Escoda-Teigell L, Martínez-González S, Turégano-Fuentes P, Requena-Caballero L (2011). Multiorgan histiocytosis after B-cell acute lymphoblastic leukemia. Am J Dermatopathol.

[12] Magni M, Di NM, Carlo-Stella C, Matteucci P, Lavazza C, Grisanti S, Bifulco C, Pilotti S, Papini D, Rosai J, Gianni AM (2002). Identical rearrangement of immunoglobulin heavy chain gene in neoplastic Langerhans cells and B-lymphocytes: evidence for a common precursor. Leuk Res.

[13] Schmitt-Graeff AH, Duerkop H, Vollmer-Kary B, Haxelmans S, Nitschke R, Fisch P, Germing U, Stein H (2012). Clonal relationship between langerhans cell histiocytosis and myeloid sarcoma. Leukemia.

[14] Yohe SL, Chenault CB, Torlakovic EE, Asplund SL, McKenna RW (2014). Langerhans cell histiocytosis in acute leukemias of ambiguous or myeloid lineage in adult patients: support for a possible clonal relationship. Mod Pathol.

[15] Chen W, Lau SK, Fong D, Wang J, Wang E, Arber DA, Weiss LM, Huang Q (2009). High frequency of clonal immunoglobulin receptor gene rearrangements in sporadic histiocytic/dendritic cell sarcomas. Am J Surg Pathol.

[16] Raj A, Bendon R, Moriarty T, Suarez C, Bertolone S (2001). Langerhans cell histiocytosis following childhood acute lymphoblastic leukemia. Am J Hematol.

[17] Egeler RM, Neglia JP, Aricò M, Favara BE, Heitger A, Nesbit ME, Nicholson HS (1998). The relation of Langerhans cell histiocytosis to acute leukemia, lymphomas, and other solid tumors. The LCH-Malignancy Study Group of the Histiocyte Society. Hematol Oncol Clin North Am.

[18] Feldman AL, Arber DA, Pittaluga S, Martinez A, Burke JS, Raffeld M, Camos M, Warnke R, Jaffe ES (2008). Clonally related follicular lymphomas and histiocytic/dendritic cell sarcomas: evidence for transdifferentiation of the follicular lymphoma clone. Blood.

[19] Cossu A, Deiana A, Lissia A, Dedola MF, Cocco L, Palmieri G, Tanda F (2006). Synchronous interdigitating dendritic cell sarcoma and B-cell small lymphocytic lymphoma in a lymph node. Arch Pathol Lab Med.

[20] Alvaro T, Bosch R, Salvadó MT, Piris MA (1996). True histiocytic lymphoma of the stomach associated with low-grade B-cell mucosa-associated lymphoid tissue (MALT)-type lymphoma. Am J Surg Pathol.

[21] Fernandez-Pol S, Bangs CD, Cherry A, Arber DA, Gratzinger D (2016). Two cases of histiocytic sarcoma with BCL2 translocations and occult or subsequent follicular lymphoma. Hum Pathol.

[22] Ambrosio MR, De Falco G, Rocca BJ, Barone A, Amato T, Bellan C, Lazzi S, Leoncini L (2015). Langerhans cell sarcoma following marginal zone lymphoma: expanding the knowledge on mature B cell plasticity. Virchows Arch.

[23] Mehrotra S, Pan Z (2015). Fine needle aspiration cytology of histiocytic sarcoma with dendritic cell differentiation: a case of transdifferentiation from low-grade follicular lymphoma. Diagn Cytopathol.

[24] Stoecker MM, Wang E (2013). Histiocytic/dendritic cell transformation of B-cell neoplasms: pathologic evidence of lineage conversion in differentiated hematolymphoid malignancies. Arch Pathol Lab Med.

[25] Dictor M, Warenholt J, György C, Månsson I, Larsson G (2009). Clonal evolution to histiocytic sarcoma with the BCR/ABL rearrangement 14 years after acute lymphoblastic leukemia. Leuk Lymphoma.

[26] Fraser CR, Wang W, Gomez M, Zhang T, Mathew S, Furman RR, Knowles DM, Orazi A, Tam W (2009). Transformation of chronic lymphocytic leukemia/small lymphocytic lymphoma to interdigitating dendritic cell sarcoma: evidence for transdifferentiation of the lymphoma clone. Am J Clin Pathol.

[27] Shao H, Xi L, Raffeld M, Feldman AL, Ketterling RP, Knudson R, Rodriguez-Canales J, Hanson J, Pittaluga S, Jaffe ES (2011). Clonally related histiocytic/dendritic cell sarcoma and chronic lymphocytic leukemia/small lymphocytic lymphoma: a study of seven cases. Mod Pathol.

[28] Ratei R, Hummel M, Anagnostopoulos I, Jähne D, Arnold R, Dörken B, Mathas S, Benter T, Dudeck O, Ludwig WD, Stein H (2010). Common clonal origin of an acute B-lymphoblastic leukemia and a Langerhans' cell sarcoma: evidence for hematopoietic plasticity. Haematologica.

[29] Dalia S, Jaglal M, Chervenick P, Cualing H, Sokol L (2014). Clinicopathologic characteristics and outcomes of histiocytic and dendritic cell neoplasms: the moffitt cancer center experience over the last twenty five years. Cancers (Basel).

[30] Xie H, Ye M, Feng R, Graf T (2004). Stepwise reprogramming of B cells into macrophages. Cell.

[31] Anjuère F, del HGM, Martín P, Ardavín C (2000). Langerhans cells develop from a lymphoid-committed precursor. Blood.

[32] Qiu X, Zhu X, Zhang L, Mao Y, Zhang J, Hao P, Li G, Lv P, Li Z, Sun X, Wu L, Zheng J, Deng Y (2003). Human epithelial cancers secrete immunoglobulin g with unidentified specificity to promote growth and survival of tumor cells. Cancer Res.

[33] Qiu X, Sun X, He Z, Huang J, Hu F, Chen L, Lin P, You MJ, Medeiros LJ, Yin CC (2013). Immunoglobulin gamma heavy chain gene with somatic hypermutation is frequently expressed in acute myeloid leukemia. Leukemia.

[34] Liao Q, Liu W, Liu Y, Wang F, Wang C, Zhang J, Chu M, Jiang D, Xiao L, Shao W, Sheng Z, Tao X, Huo L (2015). Aberrant high expression of immunoglobulin G in epithelial stem/progenitor-like cells contributes to tumor initiation and metastasis. Oncotarget.

[35] Wang C, Xia M, Sun X, He Z, Hu F, Chen L, Bueso-Ramos CE, Qiu X, Yin CC (2015). IGK with conserved IGΚV/IGΚJ repertoire is expressed in acute myeloid leukemia and promotes leukemic cell migration. Oncotarget.

